# ECT in Huntington’s Psychosis - an unexplored therapeutic option

**DOI:** 10.1192/j.eurpsy.2025.889

**Published:** 2025-08-26

**Authors:** N. Ramalho, T. Rocha, J. F. Cunha, J. C. Moura, J. Leal, D. Seabra, I. Lopes, G. Santos, M. Rosa, A. Garcia

**Affiliations:** 1 Psychiatric and Mental Health Department, ULSAR, Barreiro, Portugal

## Abstract

**Introduction:**

Huntington’s disease (HD) is a progressive neurodegenerative disorder characterized by motor dysfunction, cognitive decline, and psychiatric symptoms. Among these psychiatric manifestations, psychosis occurs in a subset of patients, presenting significant challenges for both diagnosis and treatment. While pharmacological interventions, such as antipsychotics, are commonly used to manage psychosis in HD, they often come with limited efficacy and a high risk of adverse effects. Electroconvulsive therapy (ECT), traditionally employed in the treatment of severe mood disorders and treatment-resistant psychosis, has garnered minimal attention as a therapeutic option for psychosis associated with HD. This is proven by the absence of literature focusing specifically on the use of ECT for treatment of Huntington’s Psychosis. This underexplored avenue holds potential, given ECT’s neuroplastic and neurochemical effects, which may counteract the neurodegenerative processes seen in HD. Exploring the efficacy of ECT in HD-associated psychosis could not only provide symptom relief but also offer insights into the broader neuropsychiatric management of the disease.

**Objectives:**

This review aims to highlight the therapeutic potential of ECT as a novel intervention in Huntington’s psychosis, addressing the current gap in clinical research and therapeutic strategies.

**Methods:**

A non-systematic review of the published literature using the PubMed/MEDLINE database with the MESH terms “huntington,” “psychosis” and “ECT” was made. The articles were selected according to relevance.

**Results:**

There were found 14 relevant publications that adress the use of ECT in HD. 11 of them were case reports and 3 were case series. Most of the studies show good results from the use of ECT in HD. There was improvement in several areas of the patient’s mental state, namely depressive symptoms, irritability, psychotic symptoms and psychomotor agitation.

Few case studies reported worsening of the clinical picture, namely aggravation of the catatonic symptoms or cognitive impairment.

**Conclusions:**

The limited but promising evidence from case reports and case series suggests that ECT may be an effective therapeutic intervention for addressing psychiatric symptoms, including psychosis, in patients with Huntington’s disease. The majority of studies demonstrate positive outcomes. However, the findings are not universally positive, with a few reports noting potential worsening some symptoms. Given the complexity of managing psychiatric symptoms in HD, ECT presents itself as a valuable treatment option, particularly when pharmacological approaches prove ineffective or poorly tolerated. Nonetheless, the absence of larger, controlled studies on ECT for HD psychosis underscores the need for further research to validate its safety and efficacy.

**Disclosure of Interest:**

None Declared

